# Using the World Health Organization's 4S-Framework to Strengthen National Strategies, Policies and Services to Address Mental Health Problems in Adolescents in Resource-Constrained Settings

**DOI:** 10.1186/1752-4458-5-23

**Published:** 2011-09-16

**Authors:** Jane RW Fisher, Meena Cabral de Mello

**Affiliations:** 1 Jean Hailes Research Unit, Department of Public Health and Preventive Medicine, Monash University, Melbourne Australia; 2 Department of Child and Adolescent Health and Development, World Health Organization, Avenue Appia, Geneva, Switzerland

## Abstract

**Background:**

Most adolescents live in resource-constrained countries and their mental health has been less well recognised than other aspects of their health. The World Health Organization's 4-S Framework provides a structure for national initiatives to improve adolescent health through: gathering and using strategic information; developing evidence-informed policies; scaling up provision and use of health services; and strengthening linkages with other government sectors. The aim of this paper is to discuss how the findings of a recent systematic review of mental health problems in adolescents in resource-constrained settings might be applied using the 4-S Framework.

**Method:**

Analysis of the implications of the findings of a systematic search of the English-language literature for national strategies, policies, services and cross-sectoral linkages to improve the mental health of adolescents in resource-constrained settings.

**Results:**

Data are available for only 33/112 [29%] resource-constrained countries, but in all where data are available, non-psychotic mental health problems in adolescents are identifiable, prevalent and associated with reduced quality of life, impaired participation and compromised development. In the absence of evidence about effective interventions in these settings expert opinion is that a broad public policy response which addresses direct strategies for prevention, early intervention and treatment; health service and health workforce requirements; social inclusion of marginalised groups of adolescents; and specific education is required. Specific endorsed strategies include public education, parent education, training for teachers and primary healthcare workers, psycho-educational curricula, identification through periodic screening of the most vulnerable and referral for care, and the availability of counsellors or other identified trained staff members in schools from whom adolescents can seek assistance for personal, peer and family relationship problems.

**Conclusion:**

The predominant endorsed action is not that dedicated mental health services for adolescents are required, but that mental health care should be integrated using cross-sectoral strategies into the communities in which adolescents live, the institutions they attend and the organisations in which they participate.

## Background

Adolescents constitute nearly 20% of the world's population and 85% live in the world's resource-constrained low- and lower-middle income countries [LALMIC]. Adolescence is a life phase of rapid developmental change. Developmental progress is governed by the quality of interactions between adolescents and their families, schools, communities and the broader social and cultural environment[1]. These can promote optimal development but, conversely, can also contribute to mental health problems. Most adolescents have good mental health. They are able to relate well to their families, schools, workplaces, friends and social organisations and to grow and develop emotionally, socially and physically. They feel valued, experience a growing sense of competence and autonomy, are able to form and sustain relationships and have a hopeful view of the future. However, adolescence is also a life phase when mental health problems make a significant contribution to morbidity and mortality[1-3]. If unrecognised, these render them vulnerable to poor psychological functioning in the immediate and longer term.

The nature, prevalence and universal or culture-specific determinants of mental health problems in adolescents in resource-constrained settings are now receiving some research attention[4], but it is agreed that they are under-recognised in most of these countries[1,3,5,6]. There are conceptual debates about the nature and preferred descriptors of states of poor mental health in adolescents, and disparate cultural beliefs about what constitutes normal or abnormal behaviour[7]. Recognition of mental health problems is improved through the use of descriptions of behaviours that are universally recognisable, rather than diagnostic labels which can be culture specific. The less specific term mental health problems includes these, but also incorporates common experiences like grief and adjustment to life- threatening experiences that interfere with learning and development and might be recognised by primary health care workers, but do not necessarily require specialist psychiatric care[7].

Strategies to promote optimal mental health and reduce mental disorders in adolescents in resource-constrained settings are as yet under-developed. The World Health Organization's '4-S Framework' for strengthening health sector responses for adolescent health and development provides a structure to initiatives to improve adolescent heath through: gathering and using strategic information; developing supportive, evidence-informed policies; scaling up the provision and utilization of health services; and strengthening action and linkages with other government sectors[8]. By using the 4S Framework for promoting and improving the mental health of young people, there are added opportunities to strengthen the health sector's response to related adolescent health problems of public health importance including sexual and reproductive health, HIV/AIDS, non-communicable diseases, substance dependence, nutrition, and exposure to violence.

The aim of this paper is, on the basis of a systematic review of the available evidence[9]: to outline how national policies, strategies, and services to address mental health needs of adolescents in resource constrained countries can be strengthened using the World Health Organization's 4-S Framework[8].

## Method for identifying the available evidence

The evidence retrieval process for the systematic review was undertaken in accordance with the WHO Handbook for Guideline Development and is described in full in Fisher, Cabral de Mello, Izutsu, Vijayakumar, Belfer and Omigbodun [2011][9]. No existing guidelines about or systematic reviews of the evidence regarding the identification, prevention or treatment of common mental health problems in adolescents in low or lower middle income countries were found.

Even in resource-constrained settings episodes of psychotic illness, involving florid disturbances of thinking, affect and behaviour are likely to be noticed, and to elicit help-seeking by caregivers. Developmental disabilities may have psychological consequences, but require specialist health care. This review was limited to the most common, but generally less well-recognised mental health problems reported among adolescents internationally: depression and other mood disturbance; conduct and substance abuse disorders as they co-occur with these; suicidal behaviours; mental health aspects of sexual and reproductive health, and psychological aspects of physical illness[10].

The search was limited to studies of people aged from 10 - 19 years from World Bank defined low-income [gross national income [GNI] per capita ≥ USD 935] [54 countries] and lower-middle income [GNI per capita USD 936 - 3705] [58 countries] [11], published in English or with English language abstracts. The electronic databases PubMed, Medline, PsycINFO and Embase were searched for publications from 1985 to June 2008.

Separate searches were undertaken to identify specific trials or evaluations of interventions to prevent or treat mood disorders, suicidal behaviours; psychological aspects of non-communicable and communicable health conditions and, because a literature in the field was identified, of armed conflict and natural disasters in adolescents. In addition, the papers describing epidemiological data were read to identify whether interventions to prevent or to treat common mental health problems in adolescents in these countries were described or referred to.

## Summary of the available evidence

In total, 116 articles met inclusion criteria and were included in the review[9]. Overall, data had been collected by 147 different psychometric instruments, not all locally validated, and there was little consistency in choice of measures, including those assessing comparable conditions. There was also substantial diversity in the age[s] of informants, study locations, sampling strategies, assessment of risk and protective factors and reporting and interpretation of data[9].

Investigations of the prevalence, determinants and interventions are available from 33 of the world's 112 low- and lower-middle income countries [LALMIC], but there are very few studies [≥ 3] from most of these [24/33 = 73%]. The countries which have the most data are China and India and to a lesser extent some African and Latin American countries [see Table 1]. Overall, most [70.5%] of the world's resource-constrained countries have no data about the epidemiology of adolescent mental health problems or interventions to address these needs published in the international English-language peer reviewed literature[9].

**Table 1:** Evidence regarding prevalence, nature and interventions to address mental health problems in adolescents in resource-constrained settings

Country	Mental health morbidity in community or clinical cohorts	Depressive and other emotional symptoms	Suicide and suicidal behaviours*	Mental health after armed conflict and natural disasters	Mental health consequences of non-communicable and communicable diseases	Mental health and sexual and reproductive health**	Emerging mental health concerns	TOTAL
Bangladesh		1			1	1		**3**

Bosnia				6				**6**

Cambodia				1				**1**

Cameroon						1		**1**

China		6	6			1	3	**16**

Colombia	1	2					1	**4**

Cuba				1				**1**

Ecuador						1	1	**2**

Egypt		2	1			1	1	**5**

El Salvador			1					**1**

Eritrea				1				**1**

Ethiopia		1			1	1		**3**

Gaza Strip and West Bank	1			5				**6**

Guatemala		1						**1**

Honduras				1				**1**

India	2	3	3	6	2	1	3	**20**

Iran	1	2						**3**

Iraq				3				**3**

Kosovo				1				**1**

Micronesia			1					**1**

Nepal		1		1		1		**3**

Nicaragua			2	1				**3**

Nigeria	1	5	1		3	2		**12**

Pakistan	1					2		**3**

Philippines		2						**2**

Senegal						1		**1**

Sudan				2				**2**

Sri Lanka		1	1	1				**3**

Thailand		1	1	2				**4**

Uganda		1		2	2			**5**

Ukraine		2						**2**

Zambia			1		1			**2**

Zimbabwe					2			**2**

**TOTAL**	**7**	**31**	**18**	**34**	**12**	**12**	**9**	**123**

*excluding the WHO SUPRE-MISS study, which included, but was not specific to adolescents

Source: Fisher J, Cabral de Mello M, Izutsu T, Vijayakumar L, Belfer M, Omigbodun O. Adolescent mental health in resource-constrained settings: A review of the evidence. Int J Soc Psychiatry. 2011;57[S1] 9-116.

## FIRST "S" OF WHO'S '4-S FRAMEWORK': STRATEGIC INFORMATION

The available evidence about the nature, prevalence and determinants of non-psychotic mental health problems in adolescents constitute current strategic information. It can be most readily considered in broad conceptual categories: mental health morbidity in community or general clinic attending populations; depressive and other emotional symptoms; suicide and suicidal behaviours, mental health aspects of non-communicable and communicable health conditions; sexual and reproductive health problems and exposure to armed conflict and natural disasters [see Table 2].

**Table 2:** Summary of studies about prevalence and determinants of mental health problems in adolescents in resource-constrained settings 1985-2008

Prevalence of mental health morbidity in community or clinical cohorts	7
Depressive and other symptoms of emotional disorders	30
Suicide and suicidal behaviours	18
Mental health consequences of armed conflict and natural disasters	28
Mental health consequences of non-communicable and communicable diseases	12
Mental health aspects of sexual and reproductive health	12
Emerging mental health concerns	9

Source: Fisher J, Cabral de Mello M, Izutsu T, Vijayakumar L, Belfer M, Omigbodun O. Adolescent mental health in resource-constrained settings: A review of the evidence. Int J Soc Psychiatry. 2011;57[S1] 9-116.

### Existing evidence

#### Mental health morbidity in community or clinical cohorts

There are only a few systematic surveys of general mental health morbidity in adolescents in resource-constrained countries, none representative of the whole population and none which used diagnostic measures. Prevalence of scores on self-report screening measures indicating psychiatric caseness varied by setting from 9.4% to 24%, with higher rates in girls than boys[9]. Lower estimates were found in studies which included children and did not stratify by developmental stage, and in which mood or substance disorders were not assessed so are probable underestimates[12]. Risks were poverty, low education in the adolescent or their parents, and exposure to violence or parental psychiatric illness.

A second group of studies described mental health problems in adolescents referred to specialist clinics. The data suggest that these are only accessible to those with the most severe and disabling conditions. For example, among 127 children and adolescents referred to a psychiatric clinic in Ibadan, Nigeria over a three-year period [mean age 12.07 years, 62% males], 22.8% had a psychotic disorder, 11.8% autism, 11% conduct disorder and 1.6% suicidal behaviour. Most had significant psychosocial stressors including fragmentation in family of origin and inadequate caregiving, childhood sexual or physical abuse, or parental psychiatric illness [13]. Of 150 children aged 6 to 13 years, referred to health clinics in the Gaza Strip there was a high prevalence [up to 70%] of psychological morbidity, most usually post-traumatic stress and other anxiety disorders[14]. In addition to exposure to armed conflict, parental unemployment or psychiatric or criminal history and living in crowded conditions were problematic for many of their families.

#### Depressive and other emotional symptoms

Evidence about depressive and other emotional symptoms in adolescents is available for 15/112 [13%] resource-constrained countries[9]. Most has been generated in self-report surveys incorporating psychometric instruments in groups of school attendees, some from consecutive cohorts attending health services and household surveys, but little from adolescents who are not at school. Most studies investigated associations and did not report prevalence, but all found evidence of current depressive or other emotional symptoms including: low mood, loss of interest and pleasure, hopelessness, decreased energy, social withdrawal, sleep disturbance and social and performance anxieties; or non-specific depressive equivalents including back aches, abdominal pain, headaches and dizziness and concluded that these were common, and multifactorially determined.

Diverse psychometric instruments and cut-off points were used and, where estimated, prevalence rates of clinically significant depressive symptoms ranged from 12.8% of girls and 7% of boys in Alexandria, Egypt[15] to 57.7% of young Sri Lankans[16]. Assessments of less specific symptoms included that of 205 7th grade Chinese students, 29% of the girls and 17% of the boys had felt hopeless in the past week[17]; of 339 15 year olds in Guatemala, 50% were experiencing hostility, anxiety and or depression[18]; and of 97 Ukrainian street children, 70% had emotional difficulties[19].

Depressive and other emotional symptoms in adolescents appear to be multifactorially determined. Rates were higher among those who were living in situations of socio-political disorder and civil strife or who were exposed to neighbourhood violence. Similarly they were higher in groups experiencing deprivation including poverty, homelessness, food insufficiency and malnutrition; exploitation in the labour market or who had been exposed to sexual, physical and emotional abuse. Adverse aspects of the school environment including experiences of bullying or corporal and other humiliating punishments; under recognition of learning difficulties and sensory losses and criticism of poor academic performance were more common among those with than without symptoms. Similarly, poor family functioning including parental separation and divorce; inadequate caregiving and low emotional attachment; family violence and parental mental illness and substance misuse were reported more frequently by symptomatic than asymptomatic groups. In some settings students with symptoms reported unrealistic family expectations for academic achievement. Rates were higher among girls in settings in which gender discrimination including differential and derogatory treatment of females; restricted opportunities for girls to participate in education and blame, shame and guilt associated with female sexuality were the norm[9].

#### Suicide and suicidal behaviours

Suicide is a leading cause of mortality internationally. At least 70,000 adolescents commit suicide each year, and suicide is now one of the three leading causes of death among both women and men aged 15-44 years[20]. National data about suicide do not exist in most LALMIC. There is under-reporting due to inefficient civil registration systems, variation in coroner's practices, religious sanctions and social stigma and in some settings suicidal behaviours are illegal. Evidence about suicide and suicidal behaviours in adolescents is available from 10/112 [9%] of these resource-constrained countries[9].

The main approach has been to survey large cohorts of secondary school students to assess experiences of suicidal ideas, plans or attempts to commit suicide, and correlates of these, using standardised, translated and culturally adapted survey instruments, supplemented by specific questions about family, educational, social and economic circumstances[21-25]. All had large samples of at least 950 participants who were aged on average 15.6 years. Suicidal behaviours, including thoughts of death, a 'death wish', contemplation of suicide, planning for suicide or acts of self-harm in the previous year were reported by between one in three and one in eight of these adolescents. In all these investigations both suicidal thoughts and acts of self harm were significantly more common in girls than in boys. These experiences and behaviours were associated with similar risk factors to those for depressive symptoms including: civil unrest and political instability; poverty; family dysfunction including parental divorce, absent fathers and interpersonal violence; childhood physical and sexual abuse; social isolation; academic failure and humiliation; younger age of marriage, arranged marriages, forced marriage, parental opposition to a 'love-partner' of choice, and pregnancy out of wedlock. Ease of access to lethal means of self harm in particular agricultural chemicals; limited access to primary health facilities and emergency-care hospital services and no or very low access to mental health services contribute to suicide in these contexts.

#### Mental health and psychosocial consequences of armed conflict and natural disasters

Catastrophic events including armed conflict and natural disasters affect large numbers of children and adolescents around the world[26]. In recent decades, it is estimated that twenty million children and adolescents have been displaced to refugee camps and over one million orphaned[27]. Thousands of adolescents are affected directly by natural disasters including recent cyclones, earthquakes and the Asian tsunami[28]. People living in resource-constrained countries are more likely to experience natural disasters and armed conflict than those living in high income settings [26].

Compared to children, who tend to be more sheltered by family members, adolescents are more often directly exposed to the traumatic events. Evidence about the psychosocial consequences of armed conflict and natural disasters is available from 13/112 [12%] of the world's resource-constrained countries. Twenty-eight studies have investigated the mental health consequences of armed conflict or natural disasters in either community, clinical or refugee cohorts, either limited to or including adolescents aged 10 to 19 years in resource-constrained settings: 19 pertained to armed conflict; eight to natural disasters and a single study investigated the mental health of child soldiers.

Adolescents reported high levels of exposure to traumatic events, and moderate to acute stress reactions after war, conflict or natural disaster. Symptoms of psychological distress are to be expected in the early adjustment phase after a disaster and do not represent manifestations of a disorder. There is a wide range of estimates of acute mental health problems in adolescents after natural disasters [10% to 81.8%][9]. Between a third and a half of groups of adolescents who were surveyed at varying intervals after exposure to war or armed conflict had clinically significant symptoms of post-traumatic stress disorder, anxiety, depression and non-specific somatic symptoms. Most mental health problems settle with the restoration of family and community functioning, but there are vulnerable sub-groups who have more severe and persistent mental health problems. These include those experiencing displacement, loss of family members, economic difficulties, inability to access basic daily life needs and continuing conflict. Risk appears to be heightened among those whose families functioned poorly prior to the disaster. There are particular difficulties for orphans, adolescents having to assume parenting roles and for child soldiers being repatriated from active combat.

#### Mental health aspects of non-communicable and communicable conditions

Adolescents' health and physical development are more likely to be adversely affected by chronic conditions, malnutrition, infectious diseases, environmental exposures and untreated disability in resource-constrained than high income countries. They are less likely to have access to high quality health services for treatment of illness and to rehabilitation or early intervention programs. Poor health can compromise capacity to participate in normal educational and social activities and alter developmental course, especially identity formation, which has adverse consequences for social and emotional functioning. Evidence about the mental health aspects of non-communicable and communicable conditions in adolescents is available from 7/112 [6%] of the word's LALMIC. It relates to a limited group of non-communicable [epilepsy, juvenile rheumatoid arthritis and thalassaemia] and communicable [HIV/AIDS and leprosy] health conditions. Inadequate understanding of cause can lead to pejorative misattributions, for example that epilepsy is infectious or caused by supernatural forces. Symptoms of elevated anxiety and depression, including hopelessness, despair and chronic sadness are apparent in high proportions of adolescents with non-communicable and communicable conditions. These are worse if the condition is permanent and symptoms cause disability or restrict participation.

#### Mental health aspects of sexual and reproductive health

Some of the most marked developmental changes in adolescence are in sexual identity, capacity for sexual intimacy and reproductive potential. Adolescents are particularly vulnerable because of restrictions on the provision of contraception to unmarried women; insufficient maturity and skills to negotiate safe sex and lack of access to adolescent-friendly health services. Frank discussion of sexual matters in households, schools and publications for adolescents is limited by personal embarrassment, and also by conservative social mores and religious prescriptions, leaving many with insufficient knowledge and skills to manage sexual relationships. Overall, there is high potential for mental health to be compromised by sexual and reproductive health problems and for mental health conditions to render adolescents vulnerable to sexual and reproductive health risks.

There were twelve studies which addressed mental health aspects of sexual and reproductive health in adolescents in eleven resource-constrained countries. These found that despair and suicidal behaviours are common in adolescent survivors of childhood sexual abuse or sexual violence and that adolescent marriage is a risk factor for depression in girls. Trafficked female sex workers in Nepal had high rates of symptoms of depression [100%], anxiety [97.7%] and posttraumatic stress disorder [PTSD] [29.5%][29]. In Senegal 80% of women who had experienced female genital mutilation before the age of 14 met current criteria for depression and anxiety and 30.4% for PTSD compared to 5% of women who had not been circumcised[30]. Of women waiting for surgery to repair obstetric fistulae in Dhaka, Bangladesh and Addis Ababa, Ethiopia, almost all [97%] met criteria for psychiatric caseness, 38% had major depression including active suicidal thoughts [31]. Postnatal depression and anxiety were observed in 36% of adolescent mothers in Pakistan[32] and 68% in Ecuador[33].

#### Emerging concerns in the mental health of adolescents

Globalisation and mass communications increase the likelihood that adolescents in all countries are exposed to ideas, behaviours and information that were once geographically confined. These confer benefits of increased knowledge, but are also thought to increase risk of mental health problems. There is evidence that eating disorders, and excessive concern about appearance, are emerging among adolescents in resource-constrained settings. Nine papers, including case reports and large scale surveys of school groups, had collected data from adolescents in five countries. Although rates of eating disorders were low, perceptions of being overweight were reported by up to 40% of secondary school students in China and India. There are higher rates of depressive symptoms and social adjustment difficulties in adolescents who perceive themselves as being overweight than those who regarded themselves as of normal or low weight. Other appearance concerns including that skin colour is too dark or muscle mass insufficient were attributed to aggressive marketing of cosmetic products to whiten skin and promote muscle bulk [9].

#### Evidence gaps

In many countries, some data on adolescent mental health are gathered in research studies, national or sub-national surveys, and in established health information systems, but the results and analyses are not published. The lack of accurate and up-to-date data on the mental health of adolescents hinders well-informed policy and programme formulation. While strategic published data are available for under a third of the world's resource-constrained countries, there is consistent evidence that mental health problems are apparent in adolescents in all the settings in which research has been undertaken.

## SECOND "S" OF WHO'S '4-S FRAMEWORK': SUPPORTIVE EVIDENCE-INFORMED POLICIES

National mental health strategies typically identify adolescents as an important group to consider, but rarely specify what is required to address their needs. Even when national mental health strategies contain policy statements enabling programmatic actions [e.g. they indicate that all adolescents should be provided with information about mental health] they often do not contain guiding statements informed by evidence [e.g. what the evidence base to the provision of information and education about mental health to adolescents is]. The expert clinicians and researchers in this field propose a broad public policy response which addresses prevention, identification of people with symptoms, early interventions, treatment, health system and health workforce strengthening, targeted and public education and the involvement of non-health sectors[9].

### Prevention

Effective prevention efforts require adequate national data on the nature, prevalence and determinants of mental health problems in adolescents; all causes of death including by suicide; characteristics and location of high-risk groups; and recognition of potential local resources to be mobilised. Policies about routine and targeted data collection, storage and dissemination are required.

Universal prevention strategies for mental health problems include provision of essential information about normal physical changes of adolescence; comprehensive sexual and reproductive health education and opportunities for adolescents to learn the interpersonal skills to enable them to relate with healthy assertiveness. Policies about curriculum development and teacher training are required to address these.

Specific suicide prevention policies for adolescents have to focus on risk factor reduction. This includes recognition that violence within the family [including childhood physical, verbal or sexual abuse]; the school [including bullying and harsh or humiliating punishments]; or society [including neighbourhood and community violence]; younger age of marriage, arranged marriages, forced marriage, parental opposition to a partner of choice, pregnancy when unmarried and experiences which induce shame and humiliation, including exam failure and impending punishment are associated with mental health problems and suicidal behaviours. Broad cross-sectoral policies about recognition of family and gender-based violence, the safety of adolescents at home and at school, equality of rights to education and economic participation for girls, and universal access to sexual and reproductive health care are relevant to addressing these risks.

### Early detection and intervention

Schools are vital places of connection and care for adolescents. People who interact with adolescents including teachers, sports coaches and youth workers require pre-service and in-service training to enable them to enquire about emotional experiences and needs, to respond empathically, assist with problem-solving and refer to local services if these are available. Policies about the provision of school counsellors or at least adults on the staff of schools who are identified as people, who can be approached by adolescents experiencing difficulties, are endorsed.

National disaster management policies are needed to establish that mental health interventions after emergencies should be based at community level in order to optimise the chances of being effective, affordable and culturally valid; and that interventions to treat mental health problems after disasters should only be provided for those with a confirmed psychiatric diagnosis, not to all.

### Health service and health workforce development

There are insufficient specialist mental health professionals in most LALMI countries to meet population needs. It is suggested that all health workers require both pre-service and in-service training in recognition of mental health problems in adolescents and that primary health care workers receive specific training in structured interviewing skills to enable them to ask explicitly about emotional states including depression, anxiety and suicidal ideas and behaviours. In hospitals it is proposed that health workers in emergency departments and medical wards receive advanced training in conducting psychosocial assessments of adolescents; are given structured protocols for identification of depression, substance use and common disorders related to suicidal behaviour and guidelines and plans for treatment and appropriate follow-up so as to reduce repetition of suicide attempts. Policies about curriculum development and health worker pre-service and in-service training and supervision are required to address these.

### Recognising the needs of vulnerable and marginalised groups

In all communities there are groups who are especially vulnerable either because of their circumstances or because of particular experiences or conditions. Adolescents who are living out of home, or who are working, or are not at school have less access to all resources including emotional and practical support and health care and are especially vulnerable to mental health problems.

Adolescents with chronic non-communicable and communicable conditions are also vulnerable and likely to have heightened mental health needs which warrant professional assessment and, where required, intervention. There is also high prevalence of perinatal depression and other mental health problems including suicidal behaviours among adolescent girls who become pregnant, which require assessment and psychosocial interventions which are offered in pregnancy and, continue after childbirth.

The interrelationships among exposure to violence and both mental health and sexual and reproductive health problems in adolescents mean that these exposures and conditions should be addressed together in comprehensive adolescent-friendly or adolescent-specific health services and maternity health care. National policies are required to ensure that health programmes for vulnerable and marginalised groups of adolescents incorporate mental health.

### Education of parents and the public

Public awareness of depression and other mental health problems in adolescents is low, but increased awareness and general social support are needed to promote optimal mental health in adolescents. Public education programs are required to increase parents' understanding of the emotional needs of adolescents, the circumstances in which mental health problems including suicidal behaviours might occur, the need for empathic responses to distress, and to challenge the notion that suicide is an acceptable way of dealing with adversity or humiliations. Health education programs involving the whole family are likely to improve the quality of life for adolescents with chronic medical conditions, in particular through countering misinformation and strategies to assist anxiety management in parents of adolescents so that they can promote optimal rather than anxious adjustment in their children. National health promotion policies are required to ensure that consideration of mental health is incorporated into parenting education programs and comprehensive clinical care.

### Non-health sector actions

Non-health sector actions are essential to promote optimal mental health in adolescents. Responsible reporting of suicide in the media and controlling access to the means of self harm, including safe storage of agricultural chemicals are essential suicide prevention strategies which require national policies about implementation and monitoring. National policies can greatly aid responses to natural disasters by ensuring coordinated inter-sectoral responses. The most important strategy to reduce mental health morbidity associated with female genital mutilation [FGM] is national policies about the elimination of the practice. These can include the substitution of alternative ceremonies or rituals to mark the transition to adulthood. Policies are also required to ensure that mental health care and psychosocial support are integrated into FGM programmes.

Overall there is a need to ensure that there are evidence-informed statements in national policy and strategy documents which underline the importance of addressing adolescents and list preventing mental and behavioural problems in adolescents as key objectives. They require provision of the following interventions: information and education; opportunities to build life skills; access to health and counselling services and making the environment safer and more supportive and they require the evidence base for this guidance and how these interventions will be delivered.

## THIRD "S" OF WHO'S '4-S FRAMEWORK': SCALING UP THE PROVISION OF SERVICES

In most countries, health services are provided to the general population, including adolescents, by hospitals and clinics run by the government, non-government organizations [NGOs] and individuals and organizations in the private sector. A range of barriers hinder the use of health services by adolescents. To respond to this, in many settings, public and private health services as well as NGOs are providing health services, especially sexual and reproductive health services, which are intended to respond specifically to the needs of adolescents, and to be "friendly" to them. These initiatives are often small in scale and limited in duration.

A broad public health response which includes strategies for prevention and early intervention as well as treatment services is required. No reports of trials or evaluations of interventions to prevent mental health problems in adolescents in resource-constrained settings were found. One small randomised controlled trial of a psychological intervention to treat mood and behaviour disorders after childhood sexual abuse, five papers reporting testing or evaluation of post-disaster treatment interventions [one of which was a randomised controlled trial] and one audit of data from a post-disaster mental health service were identified. However, many of the authors of the epidemiological studies endorse actions, which constitute a body of expert opinion.

### Prevention of mental health problems in adolescents in resource-constrained settings

Most adolescents in resource-constrained settings live in poverty and do not have access to specific adolescent-friendly health services. Adults with whom adolescents have contact are less likely to recognise and seek assistance for internalising symptoms of depression and other emotional disorders than psychosis and externalising behavioural symptoms. Overall, the expert opinion was that the high prevalence of depressive and other emotional symptoms including suicidal ideas in adolescents in resource-constrained contexts requires a cross-sectoral approach which is informed by a comprehensive understanding of the multiple political, cultural, social, economic, educational and familial determinants [15,19,34] Izutsu et al[35] emphasise that as risk factors are gender-governed, interventions must be gender-informed and some must be gender specific.

### Primary health care services

Primary care services are often the only and usually the first point of contact for a young person with the health care system and therefore have a crucial role to play in recognising and responding to mental health problems, particularly for those adolescents who are not in the school system. A number of strategies are proposed so that primary health workers will be able to achieve this. These include specific training in understanding the developmental stage of adolescence; the common mental health problems that can occur during this life stage; the skills required to establish a therapeutic alliance with young people and the use of supportive counselling strategies incorporating empathic listening and a problem-solving approach. Training in structured interviewing techniques to identify depressive symptoms in those presenting with conduct, emotional or substance abuse problems, or suicidal behaviours, and refer to secondary specialist services is also recommended. Community health interventions including the use of leaders and elders to reduce family violence and thereby reduce depressive and other emotional symptoms among adolescents are also recommended[36].

### Schools

Schools are crucial locations for comprehensive mental health promotion programs and a number of strategies are endorsed. These include implementation of school policies about inclusiveness, valuing of individuals and opportunities for all students to experience mastery and success. Also the provision of education programs for parents, to increase awareness of the emotional needs of adolescents, the circumstances in which depression and other mood disorders might occur, and the need for empathic responses to distress. Training for teachers in the recognition of signs of emotional distress including symptoms of depression and anxiety and identification of a staff member to whom adolescents can turn for assistance if they are facing serious life difficulties are of value. Life skills and personal development programs, including problem-solving strategies for adolescents improve emotional literacy and awareness of rights. Routine use of systematic screening with self-report questionnaires in schools to identify adolescents with current symptoms; the provision of counselling services in schools and identifying and supporting adolescents at high risk because of family dysfunctions, school difficulties or living in abusive circumstances are endorsed [16,37].

#### Treatment of mental health problems in adolescents in resource-constrained settings

There is agreement that there are insufficient psychiatrists, in particular child psychiatrists, or other specialist mental health professionals to be able to meet the needs of adolescents with mental health problems in resource-constrained countries. It is generally agreed that undergraduate medical training is restructured to include recognition of and skilled response to mental health problems in adolescents[38]. A range of additional strategies is required. There is little evidence from randomised controlled or other trials or evaluations of interventions and most endorsed actions for the treatment of mental health problems in adolescents constitute expert professional opinion.

### Depressive and other emotional symptoms

Psychological treatments including psycho-educational strategies and cognitive behavioural therapy are useful for the treatment of adolescent depression.

### Suicide and suicidal behaviours

General propositions include that protocols for identification of depression, substance use and other common disorders related to suicidal behaviour should be implemented in hospital emergency departments[39] and that staff should be trained to conduct psychosocial assessments of suicidal intent[40]. Guidelines and plans for treatment and appropriate follow-up of people presenting with suicidal behaviours should be established in order to reduce repetition of suicide attempts[39]. Access to means of self harm, including agricultural chemicals should be controlled and national regulations for training of health care workers about the safe sale of over-the-counter medications in particular hypnotics should be implemented[39]. National policies about responsible reporting of suicide in the media should be developed and implemented.

### Exposure to armed conflict or natural disasters

It should not be presumed that adolescents who have experienced disasters will inevitably experience mental health problems. The World Health Organization [41] promotes rational care in which the need to do no harm either in the immediate or longer term is emphasised and that the use of medications and unproven therapies is to be avoided[42]. There are risks to the over-diagnosis of posttraumatic stress disorder in altering self regard and promoting victimhood and clear advantages to promoting resilience and adaptive capacity[26,42-44]. Suggested actions include that psychosocial first aid: the provision of comfort and reassurance, shelter, immediate physical care, practical problem solving and purposeful activities; assistance with reunions of family members who have been separated; linking survivors with necessary resources and facilitating a sense of agency is crucial and non-specialised[26,44,45]. Assistance to resume a new form of normal life, in particular education, recreational activities and a stable social environment is restorative[26,44]. The maintenance of cultural and religious practices including funerals promotes continuity and provides reassurance. The routine exploration of experiences either orally or through art or other indirect methods of expression is not indicated[46,47]. For those with acute psychiatric symptoms, care in the local primary health care system is indicated [26,48]. Interventions must develop across all sectors, including primary health care, religious focal points, secondary and tertiary mental health, education and social services. Trauma-specific mental health services will fail to meet the needs of war-affected children and sustainable, community-based child and adolescent mental health services that address the full range of mental health problems may be a more appropriate humanitarian intervention than a psycho-trauma service which focuses on a single diagnosis[49].

Trials of treatments include a small resource-intensive trial of individual narrative exposure therapy for adolescent Somalian refugees who had experienced very severe war events and was associated with sustained PTSD symptom reduction at nine month follow up[50]. Vijayakumar et al[51] compared a six-session psycho-educational small group program which addressed enhancement of emotional literacy, anxiety management, problem solving skills, interpersonal relationships and substance use. Compared to those who did not participate, adolescents who completed the program were less overactive, valued the expression of positive emotion and were less likely to smoke. The most vulnerable children had pre-existing difficulties or family psychopathology and required enhanced care in addition to a universal intervention. In the second controlled trial of a post-tsunami intervention, inclusive of some older adolescents but aimed at bereaved adults, Vijayakumar and Kumar[52] compared individualised support by trained volunteers to standard care. Depression and suicide attempts were reduced in the intervention group.

### Mental health aspects of communicable and non-communicable diseases

Endorsed actions to manage mental health in adolescents with non-communicable chronic conditions include routine assessment of and treatment for depressive and anxiety disorders[53,54]. Addressing misinformation, stigma and discrimination through public education strategies targeting families, schools, and community organisations, is needed to reduce marginalisation and associated anxiety and depression and improve quality of life[54]. Non-pharmacological-based pain management strategies can assist adjustment and improve psychological functioning in adolescents with Juvenile Idiopathic Arthritis[55]. HIV-infected or HIV-affected adolescents should be presumed to have mental health problems which exceed the capacity of family and friends, and will benefit from mental health care integrated into all interventions relating to HIV/AIDS[56-58]. School-based HIV prevention programs should include audiovisual materials and role playing to teach safe sex negotiating skills and reduce anxiety[59]. Income-generating opportunities and provision of direct economic support to HIV-affected families are likely to improve overall functioning including mental health[57].

### Mental health aspects of sexual and reproductive health

Expert opinions include that comprehensive sexual and reproductive health education will prevent mental health problems in adolescents in resource-constrained countries. Mental health care and psychosocial support should be integrated with sexual and reproductive health services in primary care packages, including for maternal health, HIV/AIDS, survivors of gender-based violence, survivors of trafficking and surgery for obstetric fistulae. The most important strategy to reduce mental health morbidity associated with female genital mutilation is elimination of the practice, but while it persists, mental health care and psychosocial support should be part of FGM programmes.

#### Emerging concerns in the mental health of adolescents

There are no trials of interventions to promote healthy eating behaviours and reduce concerns about appearance in adolescents in resource-constrained settings. Expert opinions included general propositions that cross-sectoral prevention strategies addressing stigma reduction; rights of all adolescents to full participation; challenging of inaccurate stereotypes about ideal appearance and the integration of support and high quality sexual and reproductive health information into the services and organisations which adolescents access, are required to improve their mental health.

In summary, ministries of health are encouraged to provide youth-friendly health services and ensure that adolescents are aware of and able to access these services. Additionally, that workers receive training in how to provide youth-friendly services and, when specific youth-friendly services exist, in how to refer adolescents to such services. It is also crucial that the unique needs of adolescents for prevention and care are considered within health sector reform efforts.

## FOURTH "S" OF WHO'S '4-S FRAMEWORK': STRENGTHENING OTHER SECTORS

The Common Agenda for Adolescent Health and Development endorsed by UNFPA, UNICEF and WHO in 1995 [60] identifies health workers as central players in endeavours to optimise the health and development of adolescents. As service providers, they have important contributions to make in helping adolescents maintain good health and recover from illness through the provision of high-quality clinical and counselling services; detection diagnosis and management of health problems and as agents of change in local communities. They have an equally important contribution to make in ensuring that other sectors become engaged and deliver evidence-based interventions for protecting and promoting adolescent health and development.

There is a consistent view that the mental health of adolescents requires complementary cross-sectoral approaches and must be informed by a comprehensive understanding of the multiple political, cultural, social, economic, educational and familial determinants. Interventions must be developed across diverse sectors, including the public media, primary health care, faith-based organisations, educational institutions, social services and secondary and tertiary mental health facilities. Inter-sectoral strategies for example providing income-generating opportunities and direct economic support are likely to improve overall functioning including mental health. There is need to ensure that such activities are evidence-based; the impact of these activities is measured and that these activities are carried out in collaboration with those of the health sector.

There is a need to make sure that government policies to address adolescent mental health promote linkages across different sectors that work with adolescents, with a focus on promotion of mental health and prevention of mental and behavioural problems. Two sectors are of particular importance. ***Schools ***can become mental health promoting organizations through policies about inclusiveness, the valuing of individuals in their diversity, promotion of cooperation, prevention of harsh and humiliating punishments, prevention of bullying and harassment, and encouraging trust between students and staff[61].

The ***media ***is of crucial importance in ensuring that published material promotes emotional literacy and solution-focused responses to life difficulties and encourages help-seeking by young people if they feel vulnerable and isolated.

We want to ensure that other sectors make the complementary actions required to provide adolescents with the information and skills they need, and the self confidence and individual/collective will they need to protect and safeguard themselves; and to engage communities to respond to protect and safe guard their adolescents.

## Discussion

The World Health Organization's 4S-Framework provides a useful structure for nations to use in addressing mental health problems in adolescents.

### First "S": Strategic information

There is marked heterogeneity in conceptualisation, sampling, data collection materials and procedures and ascertainment of risk and protective factors in the available epidemiological studies. This contributes to diverse prevalence estimates, descriptions of mental health problems, proposed models of causation and implications for interventions. Nevertheless, in all resource-constrained settings in which specific epidemiological research has been undertaken, non-psychotic mental health problems in adolescents are identifiable, prevalent and associated with reduced quality of life, impaired participation and compromised development.

High proportions of adolescents in resource-constrained settings experience sustained hopelessness, despair, social isolation, shame, anxiety and other forms of suffering and these needs require recognition and attention. As there are few data about the most vulnerable and marginalised adolescents who are not at school or living with families, the existing data are likely to be underestimates of prevalence and severity. Mental health problems reflect circumstances and are most likely to occur in adolescents who are living in poverty; exposed to family, school or neighbourhood violence; lack effective empathic parental care or a stable domestic base; are socially isolated and lack relationships with peers; have experienced or been threatened with corporal or other humiliating punishments; have no opportunities to experience mastery and success or are having to manage unrealistic academic expectations.

Each country requires the strategic information of specific epidemiological data about the prevalence and nature of mental health problems in adolescents; causative pathways and sex, age and ethnic differences[2,62]. There is therefore a need to gather, analyse, and report appropriately the best epidemiological and social science data, informed by comprehensive multifactorial models, on the incidence, prevalence, antecedents and consequences of mental health problems in adolescents. It is already possible to examine all data that is available for adolescents [10-14 years old and 15-19 year olds] and the historical trends in individual indicators. The utility of existing data is maximised by ensuring the disaggregation by age and sex of data that is routinely gathered at health facilities and in national and sub-national surveys.

### Second "S": Supportive evidence-informed policies

At present most resource-constrained countries have little local evidence for the development of supportive national policies. Nevertheless, the consistency of the evidence that is available and of professional opinion about the actions that are required provides a preliminary policy base. It is agreed that a broad public policy response which addresses direct strategies for prevention, early intervention and treatment; health service and health workforce requirements; social inclusion of marginalised groups of adolescents; parent, public and primary, secondary and tertiary education; school health policies for the promotion of social and emotional wellbeing and prevention of mental health problems.

### Third "S": Services and commodities

Strategies to address mental health problems in adolescents include public education, parent education, training for teachers and primary health workers, psycho-educational curricula, identification through periodic screening of the most vulnerable and referral for care and the availability of counsellors or other identified trained staff members in schools from whom adolescents can seek assistance for personal, peer and family relationship problems. Psychologically informed health care involves the integration of explicit considerations of mental health in the primary and sexual and reproductive health services which adolescents attend.

Each country requires evidence about effective interventions; outcomes of treatment and the cost effectiveness of treatment programs; service needs, availability and barriers and facilitators to use, from which health services can be developed [2,62].

### Fourth "S": Strengthening other sectors

The predominant endorsed action is not that dedicated mental health services for adolescents are required, but that mental health care should be integrated using cross-sectoral strategies into the communities in which adolescents live, the institutions they attend, the educational programs they receive, the media to which they are exposed and the organisations in which they participate.

## Competing interests

The authors declare that they have no competing interests.

## Authors' contributions

MCdM and JF conceptualised and conducted the analysis. JF wrote the first draft of the paper, which was then revised by both authors who read and approved the final draft.

## References

[B1] Patel V Flisher A Hetrick S McGorry P Mental health of young people: a global public-health challenge The Lancet 2007 369 9569 1302 13 10.1016/S0140-6736(07)60368-7 17434406

[B2] Rahman A Mubbashar M Harrington R Gater R Developing child mental health services in developing countries J Child Psychol Psychiatry 2000 41 5 539 46 10.1111/1469-7610.00641 10946747

[B3] Patel V Flisher A Nikapota A Malhotra S Promoting child and adolescent mental health in low and middle income countries J Child Psychol Psychiatry 2008 49 313 34 10.1111/j.1469-7610.2007.01824.x 18093112

[B4] Belfer M Setting priorities: The status of child mental health care around the world Psychiatric Times 2004 21 5

[B5] Fleitlich B Goodman R Social factors associated with child mental health problems in Brazil: cross sectional survey BMJ 2001 323 7313 599 600 10.1136/bmj.323.7313.599 11557705 PMC55573

[B6] Remschmidt H Belfer M Mental health care for children and adolescents worldwide: a review World Psychiatry 2005 4 3 147 53 16633533 PMC1414760

[B7] Belfer ML Nurcombe B Remschmidt H, Nurcombe B, Belfer M, Sartorius N, A O The Epidemiology and Burden of Child and Adolescent Mental Disorder The Mental Health of Children and Adolescents 2007 Chichester: John Wiley and Sons

[B8] World Health Organisation Strengthening the health sector response to adolescent health and development 2009 Geneva, WHO http://www.who.int/child_adolescent_health/documents/cah_adh_flyer_2010/en/index.html

[B9] Fisher J Cabral de Mello M Izutsu T Vijayakumar L Belfer M Omigbodun O Adolescent mental health in resource-constrained settings: A review of the evidence Int J Soc Psychiatry 2011 57 Suppl1 9 116 10.1177/0020764010397628 21480578

[B10] Okasha A The Presidential WPA Program on Child Mental Health World Psychiatry 2003 2 3 129 30 16946917 PMC1525109

[B11] World Bank World Bank Development Indicators 2005 http://data.worldbank.org/sites/default/files/wdi05fulltext.pdf [cited 2007 December 15th]

[B12] Srinath S Girimaji SC Gururaj G Seshadri S Subbakrishna DK Bhola P Epidemiological study of child & adolescent psychiatric disorders in urban & rural areas of Bangalore, India Indian J Med Res 2005 122 1 67 79 16106093

[B13] Omigbodun OO Psychosocial issues in a child and adolescent psychiatric clinic population in Nigeria Soc Psychiatry Psychiatr Epidemiol 2004 39 8 667 72 15300378 10.1007/s00127-004-0793-x

[B14] Thabet AAM Vostanis P Social adversities and anxiety disorders in the Gaza Strip Arch Dis Child 1998 78 5 439 42 10.1136/adc.78.5.439 9659090 PMC1717577

[B15] Afifi M Depression in adolescents: Gender differences in Oman and Egypt East Mediterr Health J 2006 12 12 61 71 17037222

[B16] Perera B Torabi MR Jayawardana G Pallethanna N Depressive symptoms among adolescents in Sri Lanka: prevalence and behavioral correlates J Adolesc Health 2006 39 1 144 6 10.1016/j.jadohealth.2005.10.013 16781980

[B17] Unger JB Li Y Anderson Johnson C Gong J Chen X Chao Yang L Stressful life events among adolescents in Wuhan, China: associations with smoking, alcohol use, and depressive symptoms Int J Beh Medicine 2001 8 1 18 10.1207/S15327558IJBM0801_01

[B18] Berganza CE Aguilar G Depression in Guatemalan adolescents Adolescence 1992 27 771 82 1471558

[B19] Kerfoot M Koshyl V Roganov O Mikhailichenko K Gorbova I Pottage D The health and well-being of neglected, abused and exploited children: The Kyiv Street Children Project Child Abuse Negl 2007 31 1 27 37 10.1016/j.chiabu.2006.07.003 17208298

[B20] World Health Organization Suicide prevention [SUPRE] 2008 http://www.who.int/entity/mental_health/prevention/suicide/suicideprevent/en/

[B21] Afifi M Depression, aggression and suicide ideation among adolescents in Alexandria Neurosciences 2004 9 3 207 13 23377429

[B22] Chen J Dunne MP Han P Child sexual abuse in China: a study of adolescents in four provinces Child Abuse Negl 2004 28 11 1171 86 10.1016/j.chiabu.2004.07.003 15567022

[B23] Liu X Tein JY Zhao Z Sandler IN Suicidality and correlates among rural adolescents of China J Adolesc Health 2005 37 6 443 51 10.1016/j.jadohealth.2004.08.027 16310121

[B24] Ruangkanchanasetr S Plitponkarnpim A Hetrakul P Kongsakon R Youth risk behavior survey: Bangkok, Thailand J Adolesc Health 2005 36 3 227 35 10.1016/j.jadohealth.2004.01.013 15737779

[B25] Springer AE Selwyn BJ Kelder SH A descriptive study of youth risk behavior in urban and rural secondary school students in El Salvador BMC Int Health Hum Rights 2006 6 3 10.1186/1472-698X-6-3 PMC1459212 16608519

[B26] Morris J van Ommeren M Belfer M Saxena S Saraceno B Children and the Sphere standard on mental and social aspects of health Disasters 2007 31 1 71 90 10.1111/j.1467-7717.2007.00341.x 17367375

[B27] Southall D Abbassi K Protecting children from armed conflict Br Med J 1998 316 6 10.1136/bmj.316.7144.1549 PMC1113196 9596589

[B28] Saylor C De Roma V La Greca AM, Silverman WM, Vernberg EM, Roberts MC Assessment of children and adolescents exposed to disaster Helping Children Cope with Disasters and Terrorism 2002 Washington, DC: American Psychological Association

[B29] Tsutsumi A Izutsu T Poudyal AK Kato S Marui E Mental health of female survivors of human trafficking in Nepal Soc Sci Med 2008 66 1841-1847 10.1016/j.socscimed.2007.12.025 18276050

[B30] Behrendt A Moritz S Posttraumatic stress disorder and memory problems after female genital mutilation Am J Psychiatry 2005 162 1000-1002 10.1176/appi.ajp.162.5.1000 15863806

[B31] Goh JT Sloane KM Krause HG Browning A Akhter S Mental health screening for women with genital tract fistulae Br J Obstet Gynaecol 2005 112 1328 30 10.1111/j.1471-0528.2005.00712.x 16101616

[B32] Hussain N Beve I Hussain M Chaudhry IB Atif N Rahman A Prevalence and social correlates of postnatal depression in a low income country Arch Womens Ment Health 2006 9 197 202 10.1007/s00737-006-0129-9 16633740

[B33] Guijarro S Naranjo J Padilla M Gutierez R Lammers C Blum RW Family risk factors associated with adolescent pregnancy: study of a group of adolescent girls and their families in Ecuador J Adolesc Health 1999 25 166 72 10.1016/S1054-139X(99)00020-8 10447044

[B34] Fekadu D Alem A Hagglof B The prevalence of mental health problems in Ethiopian child laborers J Child Psychol Psychiatry 2006 47 9 954 9 10.1111/j.1469-7610.2006.01617.x 16930390

[B35] Izutsu T Tsutsumi A Islam MA Matsuo Y Yamada HS Kurita H Validity and reliability of the Bangla Version of WHOQOL-BREF in an adolescent population in Bangladesh Qual Life Res 2005 14 1783-1789 10.1007/s11136-005-1744-z 16119189

[B36] Hindin MJ Gultiano S Associations between witnessing parental domestic violence and experiencing depressive symptoms in Filipino adolescents Am J Public Health 2006 96 4 660 3 10.2105/AJPH.2005.069625 16507722 PMC1470531

[B37] Adewuya AO Ola BA Aloba OO Prevalence of major depressive disorders and a validation of the beck depression inventory among Nigerian adolescents Eur Child Adolesc Psychiatry 2007 16 287 92 10.1007/s00787-006-0557-0 17473949

[B38] Gureje O Omigbodun OO Gater R Acha RA Ikuesan BA Morris J Psychiatric disorders in a pediatric primary care clinic Br J Psychiatry 1994 165 527 30 10.1192/bjp.165.4.527 7804668

[B39] Fleischmann A Bertolote JM De Leo D Botega N Phillips M Sisask M Characteristics of attempted suicides seen in emergency-care settings of general hospitals in eight low- and middle-income countries Psychol Med 2005 35 10 1467 74 10.1017/S0033291705005416 16164770

[B40] Kumar CT Mohan R Ranjith G Chandrasekaran R Characteristics of high intent suicide attempters admitted to a general hospital J Affect Disord 2006 91 1 77 81 10.1016/j.jad.2005.12.028 16443283

[B41] World Health Organization Caring for Children and Adolescents with Mental Disorders: Setting WHO Directions 2003 Geneva: World Health Organization

[B42] Belfer ML Caring for children and adolescents in the aftermath of natural disasters Int Rev Psychiatry 2006 18 6 523 8 10.1080/09540260601048877 17162692

[B43] Silove D Bryant RA Rapid assessments of mental health needs after disasters JAMA 2006 296 576 8 10.1001/jama.296.5.576 16882965

[B44] Williams R The psychosocial consequences for children and young people who are exposed to terrorism, war, conflict and natural disasters Curr Opin Psychiatry 2006 19 4 337 49 10.1097/01.yco.0000228751.85828.c1 16721161

[B45] Becker SM Psychosocial care for adult and child survivors of the 2004 tsunami disaster in India Am J Public Health 2006 96 8 1397 8 10.2105/AJPH.2005.064428 16809599 PMC1522118

[B46] Bolton P Bass J Betancourt T Speelman L Onyango G Clougherty KF Interventions for depression symptoms among adolescent survivors of war and displacement in northern Uganda: a randomized controlled trial JAMA 2007 298 5 519 27 10.1001/jama.298.5.519 17666672

[B47] Thabet AA Vostanis P Karim K Group crisis intervention for children during ongoing war conflict Eur Child Adolesc Psychiatry 2005 14 5 262 9 10.1007/s00787-005-0466-7 15981138

[B48] Inter-Agency Standing Committee IASC Guidelines on Mental Health and Psychosocial Support in Emergency Settings 2007 Geneva: IASC 10.1080/09540261.2022.2147420 36502397

[B49] Jones L Rrustemi A Shahini M Uka A Mental health services for war-affected children: report of a survey in Kosovo Br J Psychiatry 2003 183 540 6 10.1192/bjp.183.6.540 14645026

[B50] Onyut LP Neuner F Schauer E Ertl V Odenwald M Schauer M Narrative Exposure Therapy as a treatment for child war survivors with posttraumatic stress disorder: two case reports and a pilot study in an African refugee settlement BMC Psychiatry 2005 5 7 10.1186/1471-244X-5-7 15691374 PMC549194

[B51] Vijayakumar L Kannan GK Daniel SJ Mental health status in children exposed to tsunami Int Rev Psychiatry 2006 18 6 507 13 10.1080/09540260601037581 17162690

[B52] Vijayakumar L Kumar MS Trained volunteer-delivered mental health support to those bereaved by Asian tsunami: an evaluation Int J Soc Psychiatry 2008 54 4 293 302 10.1177/0020764008090283 18720890

[B53] Adewuya AO Parental psychopathology and self-rated quality of life in adolescents with epilepsy in Nigeria Dev Med Child Neurol 2006 48 7 4 10.1017/S0012162206001253 16780631

[B54] Adewuya AO Oseni SBA Impact of psychiatric morbidity on parent-rated quality of life in Nigerian adolescents with epilepsy Epilepsy Behav 2005 7 3 497 501 10.1016/j.yebeh.2005.07.011 16143568

[B55] Mullick MSI Goodman R The prevalence of psychiatric disorders among 5-10 year olds in rural, urban and slum areas in Bangladesh: An exploratory study Soc Psychiatry Psychiatr Epidemiol 2005 40 8 663 71 10.1007/s00127-005-0939-5 16091858

[B56] Atwine B Cantor-Graae E Bajunirwe F Psychological distress among AIDS orphans in rural Uganda Soc Sci Med 2005 61 3 555 64 10.1016/j.socscimed.2004.12.018 15899315

[B57] Foster G Makufa C Drew R Mashumba S Kambeu S Perceptions of children and community members concerning the circumstances of orphans in rural Zimbabwe AIDS Care 1997 9 4 391 405 10.1080/713613166 9337884

[B58] Sengendo J Nambi J The psychological effect of orphanhood: a study of orphans in Rakai district Health Transit Rev 1997 7 Suppl 105 24 10169639

[B59] Venier JL Ross MW Akande A HIV/AIDS-related social anxieties in adolescents in three African countries Soc Sci Med 1998 46 3 313 20 10.1016/S0277-9536(97)00153-6 9460813

[B60] World Health Organization UNFPA UNICEF Action for adolescent health: Towards a common agenda 1995 http://www.who.int/entity/child_adolescent_health/documents/frh_adh_97_9/en/

[B61] World Health Organisation Creating an environment for emotional and social well-being: an important responsibility of a health-promoting and child friendly school 2005 Geneva: WHO http://www.who.int/school_youth_health/resources/information_series/en/

[B62] Belfer ML Child and adolescent mental disorders: the magnitude of the problem across the globe J Child Psychol Psychiatry 2008 49 226 36 10.1111/j.1469-7610.2007.01855.x 18221350

